# Clinical effect of *DAPK* promoter methylation in gastric cancer: A systematic meta-analysis

**DOI:** 10.1097/MD.0000000000005040

**Published:** 2016-10-28

**Authors:** Wenzhuo Jia, Tao Yu, Xianglong Cao, Qi An, Hua Yang

**Affiliations:** Department of General Surgery, Beijing Hospital, National Center of Gerontology, China.

**Keywords:** clinicopathological features, *DAPK*, GC, promoter methylation

## Abstract

**Background:**

The loss of death-associated protein kinase (*DAPK*) gene expression through promoter methylation is involved in many tumors. However, the relationship between *DAPK* promoter methylation and clinicopathological features of gastric cancer (GC) remains to be done. Therefore, we performed a meta-analysis to assess the role of *DAPK* promoter methylation in GC.

**Methods:**

Literature databases were searched to retrieve eligible studies. The pooled odds ratios (ORs) with its 95% confidence intervals (CIs) were calculated using the Stata 12.0 software.

**Results:**

Final 22 available studies with 1606 GC patients and 1508 nonmalignant controls were analyzed. A significant correlation was found between *DAPK* promoter methylation and GC (OR = 3.23, 95% CI = 1.70–6.14, *P* < 0.001), but we did not find any significant association in Caucasian population, and in blood samples in subgroup analyses. *DAPK* promoter methylation was associated with tumor stage and lymph node status (OR = 0.69, 95% CI = 0.49–0.96, *P* = 0.03; OR = 1.50, 95% CI = 1.12–2.01, *P* = 0.007; respectively). However, we did not find that *DAPK* promoter methylation was associated with gender status and tumor histology.

**Conclusion:**

Our findings suggested that *DAPK* promoter methylation may play a key role in the carcinogenesis and progression of GC. In addition, methylated *DAPK* was a susceptible gene for Asian population. However, more studies with larger subjects should be done to further evaluate the effect of *DAPK* promoter methylation in GC patients, especially in blood and Caucasian population subgroup.

## 1 Introduction

Gastric cancer (GC) is the fifth most common malignancy and the third leading cause of cancer-related deaths worldwide, especially in East Asia.^[1]^ Approximately 90% of stomach tumors are diagnosed as adenocarcinoma, which consist of 2 main histological subtypes: intestinal and diffuse.^[2]^ Although the recent diagnostic and therapeutic opportunities have supported good survival in early gastric cancer patients, numerous patients are typically diagnosed at a late stage, leading to a high mortality rate.^[3]^

Many factors are correlated with the development of gastric cancer, such as *Helicobacter pylori* (*H. pylori*) infection, dietary and lifestyle factors, genetics, and epigenetic alterations.^[^4 5^]^ DNA methylation as a major mechanism of epigenetic changes has been proven to involve in the carcinogenesis, progression, and prognosis of GC.^[^6–9^]^ The gene epigenomic regulation has 2 important components of the molecular mechanism, including the hypermethylation of tumor suppressor genes (TSGs) and hypomethylation of oncogenes.^[^10 11^]^ Death-associated protein kinase (*DAPK*), a tumor suppressor gene, encoding a calcium/calmodulin-dependent serine/threonine kinase, is localized on human chromosome 9p34 and involves in various apoptosis triggered by tumor necrosis factor-alpha (TNF-α), Fas ligand, and interferon-γ (IFN-γ).^[^12 13^]^ Moreover, *DAPK* also participates in a range of cellular processes such as growth factor signaling, inflammation and autophagy, as well as the regulation of immune responses.^[^14 15^]^ The dysfunction of *DAPK* gene via promoter methylation has been linked with many cancers.^[^16–19^]^

However, the clinical significance of *DAPK* promoter methylation in GC remains to be determined. Therefore, we performed a meta-analysis to assess the strength of the association of *DAPK* promoter methylation in GC versus noncancerous controls, in relation to sex status, tumor stage, tumor histology, and lymph node status in gastric cancer.

## 2 Materials and methods

### 2.1 Literature search

A systematic search of the literature published without language restriction was conducted on PubMed, Embase, EBSCO, and Web of Science databases prior to May 26, 2016. We used the following keywords and search terms: (stomach OR gastric) AND (cancer OR tumor OR neoplasm OR carcinoma) AND (DAPK OR death-associated protein kinase OR DAPK-kinase) AND (methylation OR epigenetic silencing OR epigenetic inactivation). This study was approved by The Institutional Review Board of Ethics Committee of Beijing Hospital.

### 2.2 Study selection

The eligibility of included studies had to meet the following criteria: (1) gastric cancer patients were histopathologically diagnosed; (2) studies were case-control design or case-series; (3) studies with sufficient information on the *DAPK* promoter methylation frequency were used to evaluate the correlation between *DAPK* promoter methylation and GC; (4) articles published as full papers in English were used in this analysis. The studies excluded did not meet inclusion criteria.

### 2.3 Data extraction

The following data from eligible studies were extracted: the first author's surname, year of publication, methylation detection methodology, ethnicity, sample type, number of methylation, the number of the case and control groups, sample size of the different histology of GC, stage of GC, gender status, and lymph node status. Tumor stages of 0–II were defined as the early stage, and tumor stages of III–IV were defined as the advanced stage.

### 2.4 Statistical analysis

The meta-analysis was performed using the Stata 12.0 software (Stata Corporation, College Station, TX). The overall odds ratios (ORs) with its 95% confidence intervals (CIs) were calculated to evaluate the strength of the correlation between *DAPK* promoter methylation and GC. The Cochran *Q* statistic and *I* ^2^ tests were conducted to test the between-study heterogeneity.^[20]^ If *I* ^2^ test shows ≥ 50%, which indicates strong heterogeneity, a random-effects model was applied; otherwise, a fixed-effects model was used.^[^21 22^]^ Egger's test was used to assess the possible publication bias.^[23]^

## 3 Results

### 3.1 General characteristics of included studies

**Figure 1:**
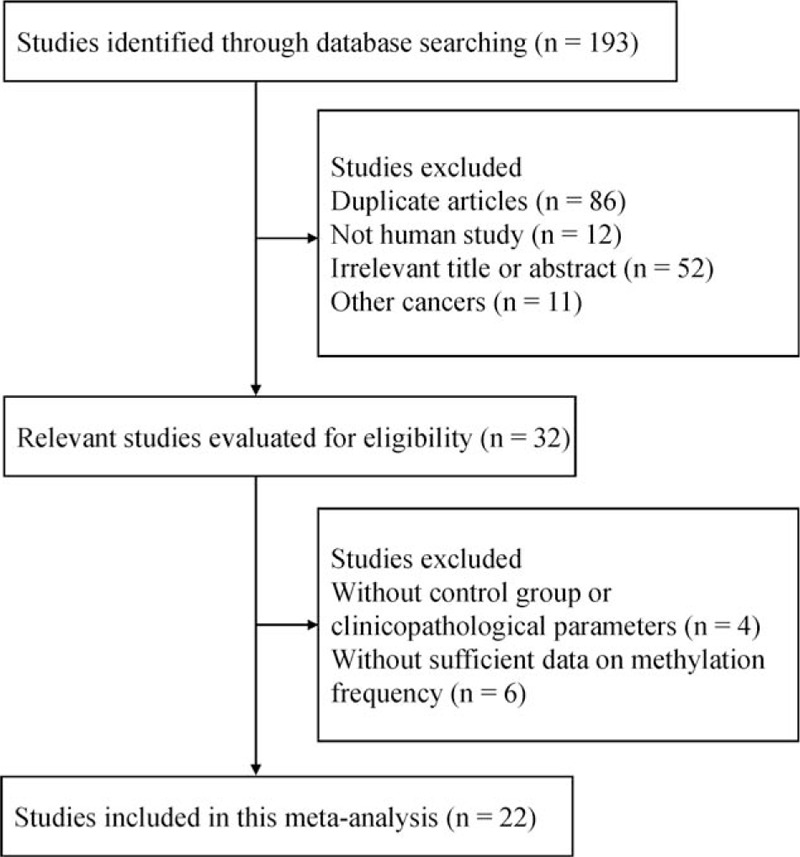
Flow diagram of the literature search strategy.

A total of 193 publications were identified by the original search as described above. Ultimately, 22 eligible studies^[^24–45^]^ involving 1606 GC patients and 1508 nonmalignant controls were included in the current meta-analysis based on our inclusion criteria (Fig. 1). Of the included studies, methylation detection methods included 1 study of combined bisulphite restriction analysis (COBRA) ^[43]^ and 21 studies of methylation-specific polymerase chain reaction (MSP).^[^24–42 44 45^]^ Eighteen studies including 1133 cases and 1508 controls assessed the *DAPK* promoter methylation status in gastric cancer and noncancerous samples.^[^24–27 29 30 33–36 38–45^]^ Fifteen studies comprising 813 male and 391 female GC patients investigated the relationship between *DAPK* promoter methylation and gender status.^[^24 25 27–29 31 32 34 36–38 40–42 44^]^ Nine studies comprising 235 early GC patients and 468 advanced GC patients investigated the association between *DAPK* promoter methylation and tumor stage.^[^24 27–29 31 32 36 38 44^]^ Twelve studies involving 607 lymph node-positive patients and 301 lymph node-negative patients investigated the association between *DAPK* promoter methylation and lymph node status.^[^24 25 27 29 32 36–38 40–42 44^]^ Nine studies involving 438 intestinal gastric cancer patients and 356 nonintestinal gastric cancer patients investigated the association between *DAPK* promoter methylation and tumor histology.^[^25 27 28 31 32 37 38 42 44^]^ The basic characteristics of included studies were presented in Table 1.

**Table 1:**
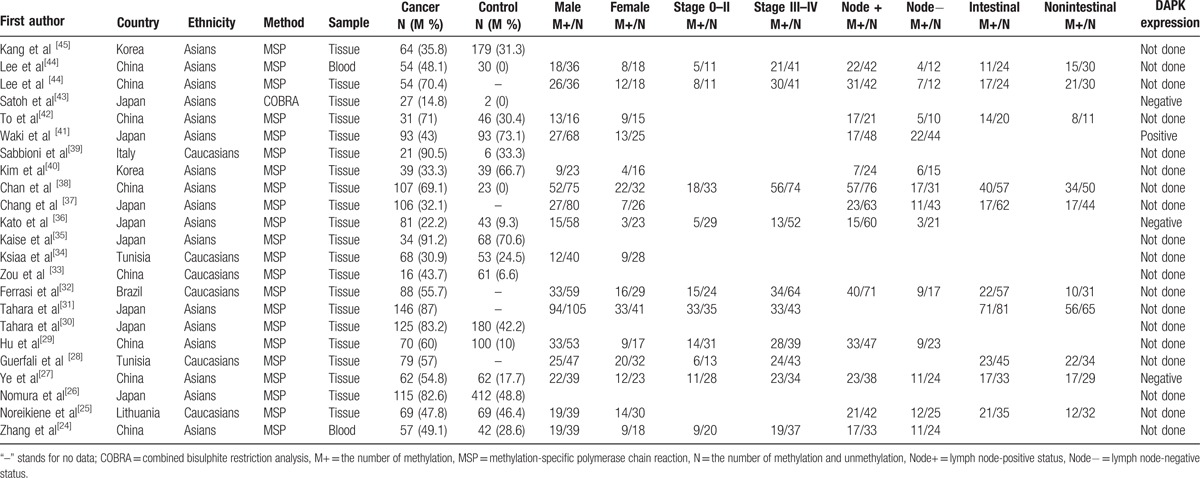
The baseline characteristics of eligible studies.

### 3.2 Pooled OR of *DAPK* promoter methylation in GC and noncancerous samples

**Figure 2:**
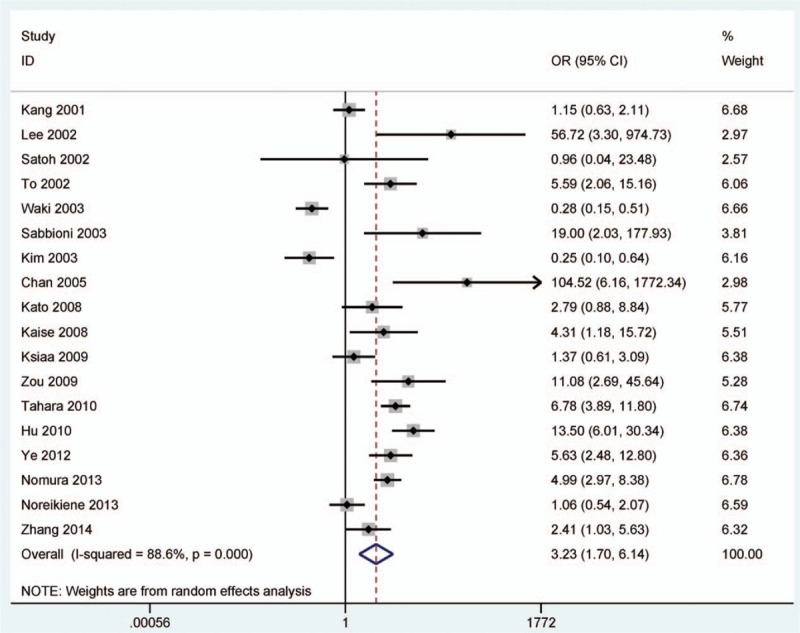
Forest plot of *DAPK* promoter methylation in GC versus nonmalignant controls. *DAPK* = death-associated protein kinase, GC = gastric cancer.

A substantial heterogeneity was found in GC and noncancerous samples (*I* ^2^ = 88.6%), the random-effects model was conducted. The result demonstrated that GC had significantly higher level of *DAPK* promoter methylation than nonmalignant samples (OR = 3.23, 95% CI = 1.70–6.14, *P* < 0.001, Fig. 2), which suggested that *DAPK* promoter methylation may play a key role in the carcinogenesis of GC.

### 3.3 Subgroup analyses

In addition, subgroup analyses were analyzed to identify the influence of *DAPK* promoter methylation on the risk of GC based on ethnicity (Asians and Caucasians) and sample type (tissue and blood) (Table 2). Subgroup analysis of ethnicity revealed that *DAPK* promoter methylation had a significant correlation with the risk of GC in Asian population (OR = 3.25, 95% CI = 1.51–7.01, *P* = 0.003), but not in Caucasian population (OR = 3.06, 95% CI = 0.96–9.70, *P* = 0.058).

**Table 2:**
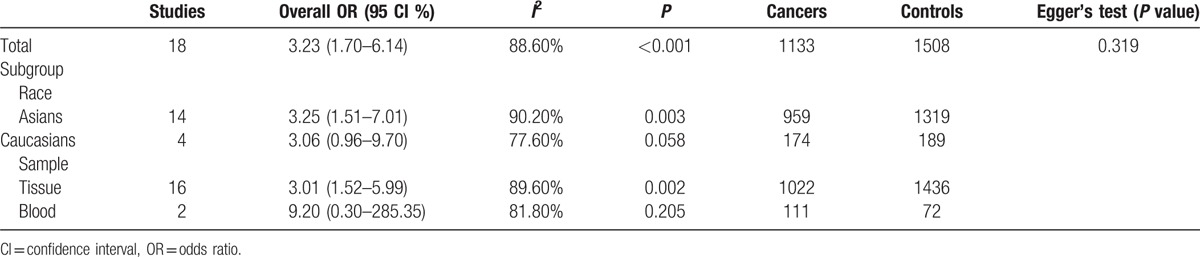
Subgroup analyses in gastric cancer versus nonmalignant controls.

Subgroup analysis of sample type showed that there was a statistical association between *DAPK* promoter methylation and increased GC risk among tissue samples (OR = 3.01, 95% CI = 1.52–5.99, *P* = 0.002), but not in blood samples (OR = 9.20, 95% CI = 0.30–285.35, *P* = 0.205). However, the result should be careful as only small subjects in blood and Caucasian population subgroup.

### 3.4 Association between *DAPK* promoter methylation and gender status in GC

As shown in Table 3, the pooled OR showed that *DAPK* promoter methylation had a similar OR in male and female under a fixed-effects model (*I* ^2^ = 0.0%, OR = 1.16, 95% CI = 0.89–1.51, *P* = 0.267), which suggested that *DAPK* promoter methylation was not associated with gender status in GC.

**Table 3:**
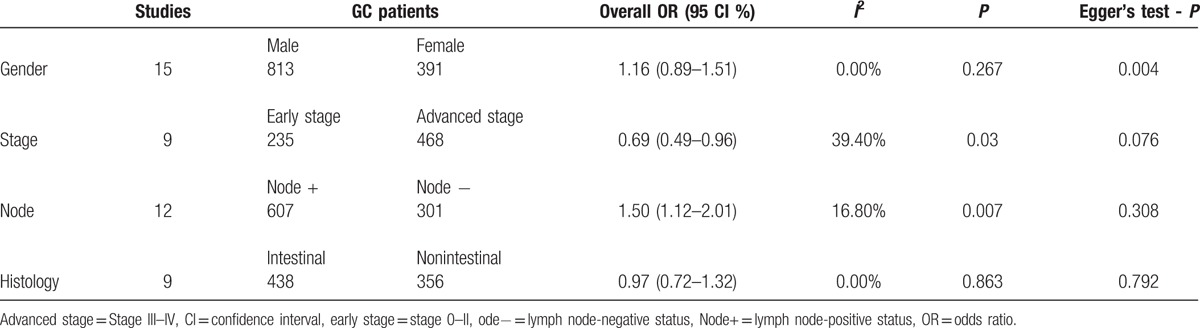
The association of *DAPK* promoter methylation and clinicopathological features.

### 3.5 Association between *DAPK* promoter methylation and tumor stage

**Figure 3:**
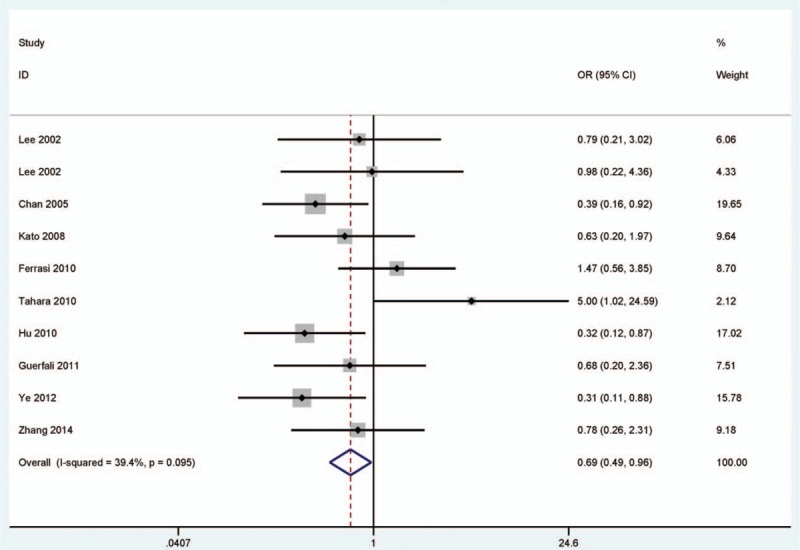
Forest plot of the association between *DAPK* promoter methylation and tumor stage. *DAPK* = death-associated protein kinase.

As shown in Fig. 3, the pooled OR revealed that *DAPK* promoter methylation was significantly lower in patients with early gastric cancer than in patients with advanced gastric cancer under a fixed-effects model (*I* ^2^ = 39.4%, OR = 0.69, 95% CI = 0.49–0.96, *P* = 0.03), which indicated that *DAPK* promoter methylation may have an increased risk in patients with advanced gastric cancer.

### 3.6 Association between *DAPK* promoter methylation and lymph node status

**Figure 4:**
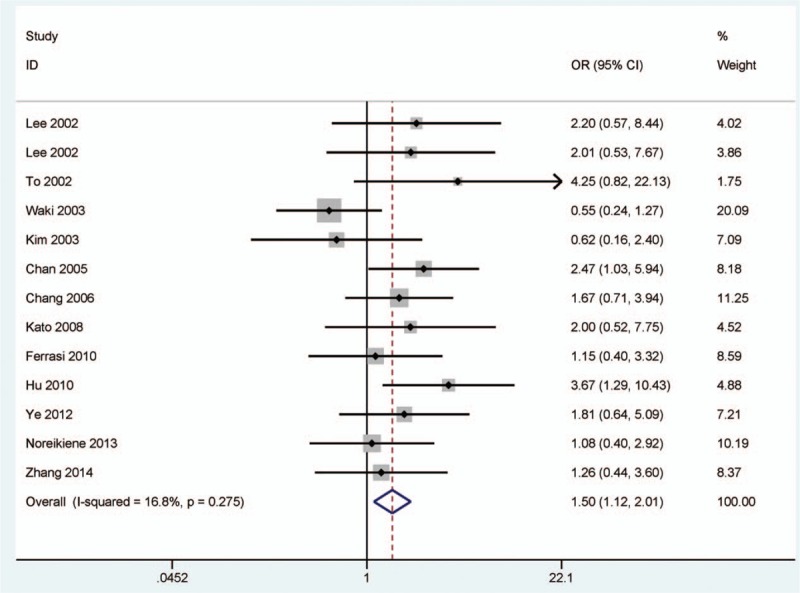
Forest plot of the association between *DAPK* promoter methylation and lymph node status. *DAPK* = death-associated protein kinase.

As shown in Fig. 4, the overall OR revealed that *DAPK* promoter methylation was significantly higher in lymph node-positive patients with gastric cancer than in lymph node-negative patients with gastric cancer under a fixed-effects model (*I* ^2^ = 16.8%, OR = 1.50, 95% CI = 1.12–2.01, *P* = 0.007), which indicated that *DAPK* promoter methylation was significantly increased risk of GC in lymph node-positive patients.

### 3.7 Association between *DAPK* promoter methylation and tumor histology

As shown in Table 3, the overall OR showed that *DAPK* promoter methylation had a similar OR in intestinal gastric cancer and nonintestinal gastric cancer under a fixed-effects model (*I* ^2^ = 0.0%, OR = 0.97, 95% CI = 0.72–1.32, *P* = 0.863), which suggested that *DAPK* promoter methylation was not correlated with tumor histology.

### 3.8 Publication bias

Egger's test was conducted to investigate the publication bias in the present analysis (Table 3). The results showed that no evidence of publication bias was observed in GC versus noncancerous controls, in relation to tumor stage, lymph node status, and tumor histology in cancer (all *P* > 0.05), and Egger's test indicated an obvious evidence of publication bias in relation to gender status in cancer (*P* = 0.004).

## 4 Discussion

The silencing or downregulation of many tumor-related genes by aberrant promoter methylation are implicated in the occurrence and development of cancers including GC.^[^14 46^]^ In this study, aberrant methylation of tumor suppressor gene *DAPK* was the CpG islands of the promoter region for all eligible studies. However, the results of *DAPK* promoter methylation were inconsistent and controversial in GC and nonmalignant tissues. Two studies reported that the frequency of *DAPK* promoter methylation was significantly lower in GC than in nonmalignant tissues.^[^40 41^]^ Three studies reported that *DAPK* promoter methylation had a similar level in GC and in nonmalignant tissues.^[^25 34 45^]^ The remaining other studies reported that *DAPK* promoter methylation had a relatively higher frequency in GC than in nonmalignant samples.^[^24 26 27 29 30 33 35 36 38 39 42–44^]^ Thus, we first determined whether *DAPK* promoter methylation was significantly correlated with the risk of GC. Next, we determined whether *DAPK* promoter methylation was associated with clinicopathological features.

Our findings revealed that the *DAPK* promoter methylation rate was significantly higher in GC than nonmalignant samples, which suggested that *DAPK* inactivation via promoter methylation plays an important in the tumorgenesis of GC. No obvious of publication bias was observed, which indicated that our result was reliable. Moreover, we conducted subgroup analyses to find the different association between *DAPK* promoter methylation and subgroups. Subgroup analysis based on ethnic population indicated that Asian population was susceptible to *DAPK* promoter methylation. Subgroup analysis based on sample type indicated that *DAPK* promoter methylation was significantly associated with the risk of GC in tissue subgroup, but not in blood subgroup, indicating that the use of *DAPK* promoter methylation as a noninvasive biomarker based on blood samples could not distinguish gastric cancer and nonmalignant samples. However, the result of blood and Caucasian population subgroup should be cautious because of small sample sizes.

To evaluate the clinical significance of *DAPK* promoter methylation in GC, we further determined whether *DAPK* promoter methylation was correlated with clinicopathological characteristics, including sex status, tumor stage, tumor histology, and lymph node status. Our results showed that *DAPK* promoter methylation was not correlated with gender status and tumor histology. However, we find that *DAPK* promoter methylation had a significant higher OR in lymph node-positive patients than in lymph node-negative patients. In addition, *DAPK* promoter methylation had a significant higher OR in advanced GC than in early GC. Our findings suggested that *DAPK* promoter methylation may involve in the occurrence and progression of GC. Egger's test showed that there was not significant publication bias in relation to tumor stage and lymph node status.

Compared with the previous meta-analysis of *DAPK* methylation status,^[47]^ our study had some differences. First, our study analyzed nonmalignant samples as the control group, including benign lesions, adjacent tissues, or normal subjects. The previous study did not involve in benign lesions (chronic gastritis and gastric adenoma). Therefore, our study suggests that *DAPK* promoter methylation plays an important role in gastric cancer initiation. Second, our study provided more studies and samples on Caucasian population (our study: 4 studies with 363 samples; the previous study: 3 studies with 273 samples). Third, our study provided more samples in relation to tumor stage (our study: 703 samples; the previous study: 567 samples), in relation to lymph node status (our study: 908 samples; the previous study: 682 samples). Thus, our result suggests that *DAPK* promoter methylation plays an important role in gastric cancer progression. Fourth, our study suggested that no significant association was found in blood subgroup with 2 studies, indicating that the use of *DAPK* promoter methylation as a biomarker based on blood samples could not distinguish GC and nonmalignant samples. The previous study only analyzed the correlation in GC versus normal blood samples. Finally, our study analyzed whether *DAPK* promoter methylation was correlated with gender status and histological type, but the previous study did not evaluated them. In addition, our study included the expression level of *DAPK* to be more informative for clinical diagnosis from *DAPK* promoter methylation to *DAPK* expression to gastric cancer incidence.

Our study had several limitations. First, although we try to search PubMed, Embase, EBSCO, and Web of Science databases as fully as possible, publication bias was found in relation to gender status in GC. Papers with other styles, such as published papers in Chinese and other languages, were excluded due to unreadable content, and unpublished studies and conference abstracts were excluded due to insufficient data. Second, only 2 studies of blood sample were included in the present study, additional studies with larger sample sizes are essential to evaluate whether *DAPK* promoter methylation can become a noninvasive biomarker for GC diagnosis in the future.

In conclusion, our findings indicated that *DAPK* promoter methylation was significantly correlated with increased risk of GC, particularly in Asian subgroup and tissue subgroup. In addition, *DAPK* promoter methylation was also associated with tumor stage and lymph node status. We did not find that *DAPK* promoter methylation was associated with gender status and tumor histology. Further large-scale studies are necessary to provide more insight into the role of *DAPK* promoter methylation in GC patients.

## References

[1] Torre LA Bray F Siegel RL Global cancer statistics, 2012. *CA Cancer J Clin* 2015; 65:87–108. 25651787 10.3322/caac.21262

[2] Crew KD Neugut AI Epidemiology of gastric cancer. *World J Gastroenterol* 2006; 12:354–362. 16489633 10.3748/wjg.v12.i3.354 PMC4066052

[3] Qu Y Dang S Hou P Gene methylation in gastric cancer. *Clin Chim Acta* 2013; 424:53–65. 23669186 10.1016/j.cca.2013.05.002

[4] Tahara T Arisawa T DNA methylation as a molecular biomarker in gastric cancer. *Epigenomics* 2015; 7:475–486. 26077432 10.2217/epi.15.4

[5] Ang TL Fock KM Clinical epidemiology of gastric cancer. *Singapore Med J* 2014; 55:621–628. 25630323 10.11622/smedj.2014174 PMC4291998

[6] Guo W Dong Z Guo Y Decreased expression and frequent promoter hypermethylation of RASSF2 and RASSF6 correlate with malignant progression and poor prognosis of gastric cardia adenocarcinoma. *Mol Carcinog* 2015; 55:1655–1666. 26456015 10.1002/mc.22416

[7] Wang K Liang Q Li X MDGA2 is a novel tumour suppressor cooperating with DMAP1 in gastric cancer and is associated with disease outcome. *Gut* 2015; 65:1619–1631. 26206665 10.1136/gutjnl-2015-309276 PMC5036270

[8] Yamanoi K Arai E Tian Y Epigenetic clustering of gastric carcinomas based on DNA methylation profiles at the precancerous stage: its correlation with tumor aggressiveness and patient outcome. *Carcinogenesis* 2015; 36:509–520. 25740824 10.1093/carcin/bgv013 PMC4417340

[9] Haam K Kim HJ Lee KT Epigenetic silencing of BTB and CNC homology 2 and concerted promoter CpG methylation in gastric cancer. *Cancer Lett* 2014; 351:206–214. 24858026 10.1016/j.canlet.2014.05.009

[10] Franco R Schoneveld O Georgakilas AG Oxidative stress, DNA methylation and carcinogenesis. *Cancer Lett* 2008; 266:6–11. 18372104 10.1016/j.canlet.2008.02.026

[11] Corson TW Gallie BL One hit, two hits, three hits, more? Genomic changes in the development of retinoblastoma. *Genes Chromosomes Cancer* 2007; 46:617–634. 17437278 10.1002/gcc.20457

[12] Kawaguchi K Oda Y Saito T Death-associated protein kinase (DAP kinase) alteration in soft tissue leiomyosarcoma: promoter methylation or homozygous deletion is associated with a loss of DAP kinase expression. *Hum Pathol* 2004; 35:1266–1271. 15492995 10.1016/j.humpath.2004.07.007

[13] Raveh T Kimchi A DAP kinase-a proapoptotic gene that functions as a tumor suppressor. *Exp Cell Res* 2001; 264:185–192. 11237533 10.1006/excr.2000.5134

[14] Huang Y Chen L Guo L Evaluating DAPK as a therapeutic target. *Apoptosis* 2014; 19:371–386. 24305735 10.1007/s10495-013-0919-2

[15] Lin Y Hupp TR Stevens C Death-associated protein kinase (DAPK) and signal transduction: additional roles beyond cell death. *FEBS J* 2010; 277:48–57. 19878313 10.1111/j.1742-4658.2009.07411.x

[16] Misawa K Mochizuki D Imai A Prognostic value of aberrant promoter hypermethylation of tumor-related genes in early-stage head and neck cancer. *Oncotarget* 2016; 7:26087–26098. 27027429 10.18632/oncotarget.8317 PMC5041966

[17] Spitzwieser M Holzweber E Pfeiler G Applicability of HIN-1, MGMT and RASSF1A promoter methylation as biomarkers for detecting field cancerization in breast cancer. *Breast Cancer Res* 2015; 17:125. 26370119 10.1186/s13058-015-0637-5 PMC4570691

[18] Arantes LM de Carvalho AC Melendez ME Validation of methylation markers for diagnosis of oral cavity cancer. *Eur J Cancer* 2015; 51:632–641. 25686481 10.1016/j.ejca.2015.01.060

[19] Kucuk C Hu X Jiang B Global promoter methylation analysis reveals novel candidate tumor suppressor genes in natural killer cell lymphoma. *Clin Cancer Res* 2015; 21:1699–1711. 25614448 10.1158/1078-0432.CCR-14-1216 PMC4504988

[20] Zintzaras E Ioannidis JP HEGESMA: genome search meta-analysis and heterogeneity testing. *Bioinformatics* 2005; 21:3672–3673. 15955784 10.1093/bioinformatics/bti536

[21] Higgins JP Thompson SG Deeks JJ Measuring inconsistency in meta-analyses. *BMJ* 2003; 327:557–560. 12958120 10.1136/bmj.327.7414.557 PMC192859

[22] DerSimonian R Meta-analysis in the design and monitoring of clinical trials. *Stat Med* 1996; 15:1237–1248.discussion 49–52. 8817798 10.1002/(SICI)1097-0258(19960630)15:12<1237::AID-SIM301>3.0.CO;2-N

[23] Egger M Davey Smith G Schneider M Bias in meta-analysis detected by a simple, graphical test. *BMJ* 1997; 315:629–634. 9310563 10.1136/bmj.315.7109.629 PMC2127453

[24] Zhang X Zhang X Sun B Detection of aberrant promoter methylation of RNF180, DAPK1 and SFRP2 in plasma DNA of patients with gastric cancer. *Oncol Lett* 2014; 8:1745–1750. 25202403 10.3892/ol.2014.2410 PMC4156173

[25] Kupcinskaite-Noreikiene R Skieceviciene J Jonaitis L CpG island methylation of the MLH1, MGMT, DAPK, and CASP8 genes in cancerous and adjacent noncancerous stomach tissues. *Medicina (Kaunas)* 2013; 49:361–366. 24509146

[26] Nomura T Tahara T Shiroeda H Influence of HRH2 promoter polymorphism on aberrant DNA methylation of DAPK and CDH1 in the gastric epithelium. *BMC Gastroenterol* 2013; 13:1. 23280118 10.1186/1471-230X-13-1 PMC3583698

[27] Ye M Li D Zhou F Epigenetic regulation of death-associated protein kinase expression in primary gastric cancers from Chinese patients. *Eur J Cancer Prev* 2012; 21:241–246. 21979297 10.1097/CEJ.0b013e32834c9caa

[28] Ben Ayed-Guerfali D Benhaj K Khabir A Hypermethylation of tumor-related genes in Tunisian patients with gastric carcinoma: clinical and biological significance. *J Surg Oncol* 2011; 103:687–694. 21308683 10.1002/jso.21875

[29] Hu SL Kong XY Cheng ZD Promoter methylation of p16, Runx3, DAPK and CHFR genes is frequent in gastric carcinoma. *Tumori* 2010; 96:726–733. 21302620 10.1177/030089161009600515

[30] Tahara T Shibata T Nakamura M Increased number of CpG island hypermethylation in tumor suppressor genes of non-neoplastic gastric mucosa correlates with higher risk of gastric cancer. *Digestion* 2010; 82:27–36. 20150736 10.1159/000252766

[31] Tahara T Shibata T Arisawa T CpG island promoter methylation (CIHM) status of tumor suppressor genes correlates with morphological appearances of gastric cancer. *Anticancer Res* 2010; 30:239–244. 20150642

[32] Ferrasi AC Pinheiro NA Rabenhorst SH Helicobacter pylori and EBV in gastric carcinomas: methylation status and microsatellite instability. *World J Gastroenterol* 2010; 16:312–319. 20082476 10.3748/wjg.v16.i3.312 PMC2807951

[33] Zou XP Zhang B Zhang XQ Promoter hypermethylation of multiple genes in early gastric adenocarcinoma and precancerous lesions. *Hum Pathol* 2009; 40:1534–1542. 19695681 10.1016/j.humpath.2009.01.029

[34] Ksiaa F Ziadi S Amara K Biological significance of promoter hypermethylation of tumor-related genes in patients with gastric carcinoma. *Clin Chim Acta* 2009; 404:128–133. 19336228 10.1016/j.cca.2009.03.044

[35] Kaise M Yamasaki T Yonezawa J CpG island hypermethylation of tumor-suppressor genes in H. pylori-infected non-neoplastic gastric mucosa is linked with gastric cancer risk. *Helicobacter* 2008; 13:35–41. 10.1111/j.1523-5378.2008.00572.x 18205664

[36] Kato K Iida S Uetake H Methylated TMS1 and DAPK genes predict prognosis and response to chemotherapy in gastric cancer. *Int J Cancer* 2008; 122:603–608. 17943730 10.1002/ijc.23143

[37] Chang MS Uozaki H Chong JM CpG island methylation status in gastric carcinoma with and without infection of Epstein–Barr virus. *Clin Cancer Res* 2006; 12:2995–3002. 16707594 10.1158/1078-0432.CCR-05-1601

[38] Chan AW Chan MW Lee TL Promoter hypermethylation of death-associated protein-kinase gene associated with advance stage gastric cancer. *Oncol Rep* 2005; 13:937–941. 15809761

[39] Sabbioni S Miotto E Veronese A Multigene methylation analysis of gastrointestinal tumors: TPEF emerges as a frequent tumor-specific aberrantly methylated marker that can be detected in peripheral blood. *Mol Diagn* 2003; 7:201–207. 15068392 10.1007/BF03260039

[40] Kim WS Son HJ Park JO Promoter methylation and down-regulation of DAPK is associated with gastric atrophy. *Int J Mol Med* 2003; 12:827–830. 14612952

[41] Waki T Tamura G Sato M Promoter methylation status of DAP-kinase and RUNX3 genes in neoplastic and non-neoplastic gastric epithelia. *Cancer Sci* 2003; 94:360–364. 12824905 10.1111/j.1349-7006.2003.tb01447.x PMC11160204

[42] To KF Leung WK Lee TL Promoter hypermethylation of tumor-related genes in gastric intestinal metaplasia of patients with and without gastric cancer. *Int J Cancer* 2002; 102:623–628. 12448005 10.1002/ijc.10783

[43] Satoh A Toyota M Itoh F DNA methylation and histone deacetylation associated with silencing DAP kinase gene expression in colorectal and gastric cancers. *Br J Cancer* 2002; 86:1817–1823. 12087472 10.1038/sj.bjc.6600319 PMC2375414

[44] Lee TL Leung WK Chan MW Detection of gene promoter hypermethylation in the tumor and serum of patients with gastric carcinoma. *Clin Cancer Res* 2002; 8:1761–1766. 12060614

[45] Kang GH Shim YH Jung HY CpG island methylation in premalignant stages of gastric carcinoma. *Cancer Res* 2001; 61:2847–2851. 11306456

[46] Tamura G Alterations of tumor suppressor and tumor-related genes in the development and progression of gastric cancer. *World J Gastroenterol* 2006; 12:192–198. 16482617 10.3748/wjg.v12.i2.192 PMC4066026

[47] Xu JN Liu ZH Shen D Clinical significance of DAPK promoter hypermethylation in gastric cancer: a meta-analysis. *Int J Clin Exp Med* 2016; 9:7883–7895.

